# Toxicity of Nanoparticles and an Overview of Current Experimental Models

**DOI:** 10.7508/ibj.2016.01.001

**Published:** 2016-01

**Authors:** Haji Bahadar, Faheem Maqbool, Kamal Niaz, Mohammad Abdollahi

**Affiliations:** 1 Dept. of Toxicology and Pharmacology, Faculty of Pharmacy and Pharmaceutical Sciences Research Center, International Campus, Tehran University of Medical Sciences, Tehran, Iran;; 2 Endocrinology and Metabolism Research Center, Institute of Clinical Endocrine Sciences, Tehran University of Medical Sciences, Tehran, Iran;; 3 Toxicology and Poisoning Research Center, Tehran University of Medical Sciences, Tehran, Iran

**Keywords:** Cytotoxicity, *in vitro*, Metal nanoparticles, Toxicology, Review

## Abstract

Nanotechnology is a rapidly growing field having potential applications in many areas. Nanoparticles (NPs) have been studied for cell toxicity, immunotoxicity, and genotoxicity. Tetrazolium-based assays such as MTT, MTS, and WST-1 are used to determine cell viability. Cell inflammatory response induced by NPs is checked by measuring inflammatory biomarkers, such as IL-8, IL-6, and tumor necrosis factor, using ELISA. Lactate dehydrogenase (LDH) assay is used for cell membrane integrity. Different types of cell cultures, including cancer cell lines have been employed as *in vitro* toxicity models. It has been generally agreed that NPs interfere with either assay materials or with detection systems. So far, toxicity data generated by employing such models are conflicting and inconsistent. Therefore, on the basis of available experimental models, it may be difficult to judge and list some of the more valuable NPs as more toxic to biological systems and vice versa. Considering the potential applications of NPs in many fields and the growing apprehensions of FDA about the toxic potential of nanoproducts, it is the need of the hour to look for new internationally agreed free of bias toxicological models by focusing more on *in vivo* studies.

## INTRODUCTION

Engineered nanoparticles (NPs) are commercially produced materials having at least one dimension less than 100 nm^[^^1^^]^. Nano-technology has brought a great revolution in the industrial sector. Due to their distinctive physicochemical and electrical properties, nano-sized materials have gained considerable attraction in the field of electronics, biotechnology, and aerospace engineering. In the field of medicine NPs are being employed as a novel delivery system for drugs, proteins, DNA, and monoclonal antibodies^[^^2^^-^^4^^]^. So far, NPs have been prepared from metal and non-metal, polymeric materials and bioceramics. The majority of NPs having medical applications are liposomes, polyethylene glycol, and dendrimers^[^^5^^]^. Humans are exposed to various nano-scale materials since childhood, and the new emerging field of nanotechnology has become another threat to human life^[^^6^^]^. Because of their small size, NPs find their way easily to enter the human body and cross the various biological barriers and may reach the most sensitive organs^[^^7^^]^. Scientists have proposed that NPs of size less than 10 nm act similar to a gas and can enter human tissues easily and may disrupt the cell normal biochemical environment^[^^8^^]^. Animals and human studies have shown that after inhalation and through oral exposure, NPs are distributed to the liver, heart, spleen, and brain in addition to lungs and gastrointestinal tract^[^^9^^-^^11^^]^. In order to clear these NPs from the body, the components of the immune system are activated. The estimated half life of NPs in human lungs is about 700 days posing a consistent threat to respiratory system. During metabolism, some of the NPs are congregated in the liver tissues^[^^6^^-^^12^^]^. NPs are more toxic to human health in comparison to large-sized particles of the same chemical substance, and it is usually suggested that toxicities are inversely proportional to the size of the NPs^[^^13^^-^^15^^]^. Due to their characteristic physicochemical properties in different biological systems, unpredictable health outcomes of NPs were eminent to scientists. So, to bridge the gap of knowledge and to exclusively tackle the toxicity issues related to NPs, different thinking aiming to contribute to safe use of NPs is felt essential.

**Nanomaterials of different substances and their toxicity**

***NPs of metallic substances***

*Aluminum oxide*

Aluminum-based NPs contribute 20% to all nano-sized chemicals. According to a report issued by “The Global Market for Aluminum Oxide NPs”, aluminum-based NPs are being used in many areas such as fuel cells, polymers, paints, coatings, textiles, biomaterials etc. (http://www.futuremarket sinc.com). About their toxic effects, Chen *et al.*^[^^16^^]^ have reported that aluminum oxide NPs disturb the cell viability, alter mitochondrial function, increase oxidative stress, and also alter tight junction protein expression of the blood brain barrier (BBB). Other researchers, for example, Radziun *et al.*^[^^17^^]^ have described that aluminum oxide NPs, at concentrations of 10, 50,100, 200, and 400 µg/mL possess no significant toxic effect on viability of mammalian cells. These investigators employed EZ4U assay technique instead of MTT for cell viability assessment. Similarly, another study has reported a dose-dependent (25-40 µg/mL) cytotoxicity as the effect of aluminum oxide NPs (160 nm) on human mesenchymal stem cells. Cell cytotoxicity in this study was also measured by MTT assay^[^^18^^]^. Besides, these NPs have been screened for genotoxicity. Balasubramanyam *et al.*^[^^19^^]^ have reported that aluminum oxide NPs (30-40 nm) possess dose-dependent genotoxic properties. They assessed genotoxicity with comet assay and micronucleus test using rat blood cells. The result of another study using mouse lymphoma cells line also suggest that aluminum oxide NPs (<50 nm) cause genotoxic effects in the form of DNA damage without any mutagenic effects^[^^20^^]^. There are very few *in vivo* studies which have reflected on this aspect of NPs. 

According to available literature, aluminum oxide NPs have been tested with main focus on cytotoxicity and genotoxic effects. Based on its huge application in various areas and subsequent human exposure, it is extremely desirable to screen aluminum-based NPs for other toxic health effects on humans according to standard protocols.

*Gold*

Gold NPs have very unique physicochemical properties. They have the capability of easy functionalization; binding to amine and thiol groups. All these characteristics possessed by gold NPs pave the way for surface modification, and are being investigated as drug carriers in cancer and thermal therapy, and as contrast agents^[^^21^^]^. Gold NPs are considered to be relatively safe, as its core is inert and non-toxic. In one experimental study, several gold NPs (4, 12, and 18 nm) with different caping agents have been investigated for any cytotoxicity against leukemia cells line^[^^22^^]^. The results of this report suggest that spherical gold NPs enter the cell and are non-toxic to cellular function. The cytotoxicity was evaluated by MTT assay^[^^22^^]^. However, there are some other reports suggesting that cytotoxicity associated with gold NPs depends on dose, side chain (cationic) and the stabilizer used^[^^23^^,^^24^^]^. Cytotoxicity of gold NPs are dependent on the type of toxicity assay, cell line, and physical/chemical properties. The variation in toxicity with respect to different cell lines has been observed in human lung and liver cancer cell line^[^^25^^]^.

*Copper oxide*

Copper oxide NPs are used in semiconductors, anti-microbial reagents, heat transfer fluids, and intrauterine contraceptive devices^[^^26^^]^. Experimentally, copper nano-materials have been documented to possess toxic effects on the liver and kidney^[^^27^^]^. Nano-copper has resulted severe impairment in liver, kidney, and spleen in experimental animals. After oral administration and interacting with gastric juice, highly reactive ionic copper is formed, which is then accumulated in the kidney of exposed animals^[^^28^^,^^29^^]^. In one *in vitro *study, copper oxide NPs (50 nm), have been reported as being genotoxic and cytotoxic along with disturbing cell membrane integrity and inducing oxidative stress^[^^30^^]^.

*Silver*

Historically, silver has long been known as an anti-bacterial substance. Its NPs are being used in a wide range of commercial products. Silver NPs are used in the form of wound dressings, coating of surgical instruments and prostheses^[^^31^^]^. They enter human body via different ways and accumulate in different organs, crossing the BBB and reach brain. Experimentally, silver NPs have been detected in various organs, including lungs, spleen, kidney, liver, and brain after exposing the rats to silver-based NPs either via inhalation or by subcutaneous injection^[^^32^^]^. Furthermore, in comparison to others, these NPs have shown more toxicity in term of cell viability, generation of reactive oxygen species (ROS), and lactate dehydrogenase (LDH) leakage^[^^33^^]^. Silver NPs are available in different coatings, each having different degrees of cytotoxicity. After exposing polyvinyl-pyrrolidone-coated silver NPs (6-20 nm) to human lung cancer cell line, Foldbjerg *et al*.^[^^34^^] ^have reported a dose-dependent cytotoxicity, and cellular DNA adduct formation. In this *in vitro* study, the MTT dye-based technique has been used for assessing the viability of human lung cancer cell line. There is one more report supporting the previously mentioned study up to some extents. These authors are of the opinion that peptide-coated silver NPs (20 nm) are more cytotoxic versus citrate-coated silver NPs of the same size. Human leukemia cell line was used in this study, and the cytotoxicity of the cells was determined by WST-1 assay^[^^35^^]^. Based on special biokinetic characteristics, as discussed earlier, it is necessary to address the toxicity-related issues of silver NPs in appropriate experimental models in light of standard protocols with respect to kidney, liver, lungs, and central nervous system disorders and most importantly in relation to endocrine functions. 

*Zinc oxide*

NPs produced from zinc oxide have many applications and are being used in paints, wave filters, UV detectors, gas sensors, sunscreens, and many personal care products^[^^36^^,^^37^^]^. On the basis of increased use in many areas, human exposure to zinc oxide NPs is imminent^[^^38^^]^. Zinc oxide NPs have been studied for any possible toxic effects on bacteria and mammalian cells. Cytotoxicity, cell membrane damage, and increased oxidative stress have been reported in various mammalian cell lines as the most common toxic effect of zinc-based nanomaterials^[^^37^^]^. After exposing human mesothelioma cells and rodent fibroblast cells to zinc oxide NPs with high concentration (49 mg/mL), Brunner* et al.*^[^^39^^] ^found almost complete cell death in the cell culture. Similarly, in another *in vitro* study, zinc oxide NPs have been accounted for change in cell morphology, DNA damage, alteration in mitochondrial activity in human hepatocytes, and embryonic kidney cells. In this experiment, MTT and comet assays have been used for measuring the cell viability and DNA damage, respectively^[^^40^^]^. Using human dermal fibroblast cells as a model and MTT as an assay technique, Meyer *et al.*^[^^41^^]^ have reported a decrease in cell viability after exposing the above cell lines to zinc oxide NPs (20 nm). Besides cytotoxicity, the genotoxic potential of zinc oxide NPs has been reported in both *in vivo* and *in vitro* studies. An *in vitro* study utilizing HEp-2 cell line, published by Osman *et al.*^[^^42^^]^ revealed that zinc oxide NPs exert its genotoxic effect via DNA damage. Standard techniques such as comet assay and cytokinesis-blocked micronucleus assay were used to determine the genotoxic potential of zinc oxide NPs in this *in vitro* study. Chronic exposure to zinc oxide NPs (300 mg/kg) resulted in oxidative DNA damage along with altering various enzymes of the liver. DNA damage was measured by using the comet assay technique^[^^43^^]^.

*Iron oxide*

Iron oxide NPs have been used in biomedical, drug delivery, and diagnostic fields. These NPs bioaccumulate in the liver and other reticuloendothelial system organs^[^^44^^,^^45^^]^. *In vivo* studies have shown that after entering the cells, iron oxide NPs remain in cell organelles (endosomes/lysosomes), release into cytoplasm after decomposing, and contribut to cellular iron poll. Magnetic iron oxide NPs have been observed to accumulate in the liver, spleen, lungs, and brain after inhalation, showing its ability to cross BBB^[^^46^^]^. Evidence show that these NPs exert their toxic effect in the form of cell lysis, inflammation, and disturbing blood coagulation system^[^^47^^]^. Also, reduced cell viability has been reported as the most common toxic effect of iron oxide NPs in *in vitro* studies. Iron oxide NPs coated with different substances have shown variable cell viability results. The toxicity of Tween-coated supermagnetic iron oxide NPs (30 nm) on murine macrophage cells has been reported by Naqvi *et al.*^[^^44^^]^. They are of the opinion that low concentration of iron oxide NPs (25-200 µg/mL for 2 h exposure) shows more cell toxicity in comparison to high concentrations (300-500 µg/mL for 6 h exposure). However, dextran-coated iron oxide NPs (100-150 nm, 0.1 mg/ mL) reduced cell viability in human macrophages by 20% after seven days of incubation^[^^48^^]^. Moreover, in another study on mouse neuroblastoma (Neuro-2A) cell line, iron oxide NPs (25 nm) have been found to exert less toxic effect in term of changing cell morphology, cell permeability, cell apoptosis, and mitochondrial function^[^^49^^]^. Using human hepatocellular carcinoma cells, chitosan-coated iron oxide NPs (13.8 nm) at concentration of 123.52 µg/mL have shown 10% cell viability after 12 h exposure^[^^50^^]^. However, 1-hydroxy-ethylidene-1,1-bisphosphonic acid coated iron oxide NPs (20 nm, 0.1 mg/mL) have shown 70% cell viability after exposing rat's mesenchymal stem cells for two days. Cell viability was determined by MTS assay in this study^[^^51^^]^. It has been thought that the toxic effects of iron oxide NPs are due to excessive production of ROS. These generated ROS further elicit DNA damage and lipid peroxidation^[^^46^^]^. 

*Titanium oxide*

Titanium oxide is chemically an inert compound, but studies have shown that NPs of titanium dioxide possess some toxic health effects in experimental animals, including DNA damage as well as genotoxicity and lung inflammation^[^^52^^,^^53^^]^. Titanium dioxide NPs (<100 nm) induce oxidative stress and form DNA adducts^[^^54^^]^. Besides genotoxicity, titanium dioxide NPs (5-200 nm) possess toxic effects on immune function, liver, kidney, spleen, myocardium, glucose, and lipids homeostasis in experimental animals^[^^55^^,^^56^^]^. 

***NPs of non-metallic substances***

*Carbon-based nanomaterials*

From application point of view, the carbon-based nanomaterials, such as carbon nanotubes, fullerenes, single and multi-walled carbon nanotubes are the most attractive and are widely used nanomaterials^[^^57^^]^. Carbon-based nanomaterials have been reported in literature as cytotoxic agents. Magrez *et al.*^[^^58^^] ^have reported that carbon-based nanomaterials possess size-dependent cytotoxicity. These investigators have tested various forms of carbon NPs on lung cancer cells to assess cell viability with MTT assay. Moreover, similar results have been published by another study conducted by Herzog *et al.*^[^^59^^] ^on carbon nanomaterials. These investigators have used a different approach for the evaluation of cell toxicity by using the clonogenic assay technique for cell proliferation and cell death. They utilized human alveolar carcinoma epithelial cell line, normal human bronchial epithelial cell line, and human keratinocytes cell line. Carbon nanotubes exert size-dependent toxicity. In animals, multi-walled carbon nanotubes have produced carcinogenic effects similar to asbestos after injecting into peritoneal cavity, as compared to single-walled carbon nanotubes, which were readily taken up by macrophages^[^^60^^]^. However, long-term accumulation of single-walled carbon nanotubes in the liver has caused disturbance in certain biochemical parameters in the form of LDH, aspartate transaminases, alanine transaminase, glutathione, and malondialdehyde along with changing the organ indices in experimental animals^[^^61^^]^. In case of carbon NPs, along with size, method of preparation and the presence of trace metals determine the extent of toxicity and biological response of the cells^[^^62^^,^^63^^]^. Fullerenes are type of carbon-based nanomaterials. They are extensively present in our environment released from fuel combustion. Non-functionalized fullerenes C60 are highly distributed in all tissues, and long-term accumulation has been observed in the liver, kidney, bones, and spleen^[^^64^^-^^66^^]^. *In vitro* studies have shown that fullerenes exert genotoxicity in the form of DNA strand breakage, chromosomal damage, and micronucleus formation after incubating fullerenes (1 ng/mL) with Chinese hamster ovary cells, human epidermoid-like carcinoma cells and human embryonic kidney cells (HEK293) for 80 days^[^^67^^,^^68^^]^. However, according to another study, fullerenes have been found with no significant effect on DNA strand breakage as determined by comet assay^[^^69^^]^. These variations in the results might be related to different experimental conditions used. The safe use of carbon NPs cannot be ascertained due to the lack of comprehensive evaluation of toxicity data. 

*Silica*

The uses of silica NPs have many advantages in drug delivery systems. silica NPs have been reported as easily functionalized drug carriers^[^^70^^]^. Besides, having application in drug delivery systems, silicon dioxide NPs are also present in ambient air comprising of 8% of all air born NPs^[^^71^^]^. Previously, nanosilica was thought as a highly biocompatible material in drug delivery systems, but according to recent reports, NPs of silica cause the generation of ROS and subsequent oxidative stress^[^^72^^]^. Lin *et al.*^[^^73^^]^ have reported an increase in the level of ROS, LDH, and malondialdehyde after treating human bronchoalveolar carcinoma cells with silica NPs (15-46 nm,) at a dosage range of 10-100 µg/mL. In this experiment, ROS has been measured with 2',7'-dichlorofluorescin diacetate, LDH, with a commercial kit. Similarly, induction of inflammatory biomarkers such as IL-1, IL-6, IL-8, TNF-α (tumor necrosis factor) and mitochondrial damage by silica NPs have been reported in various other studies^[^^74^^-^^76^^]^. In one more *in vitro* study on liver cells, silica-based NPs (70 nm) at 30 mg/kg have been found to alter biochemical parameters along with hepatotoxic effects^[^^77^^]^. 

*NPs of polymeric materials*

Biodegradable or polymeric NPs have the potential to be used in targeted drug delivery in cancer chemotherapy. These NPs are also employed in encapsulation of various molecules to develop nanomedicine providing sustained release and good biocompatibility with cells and tissues^[^^78^^]^. In addition, they have the potential to be successfully used in encapsulation of peptides, nucleic acids, and proteins. They are also considered as non-toxic, non-immunologic, non-inflammatory and do not activate neutrophils. Poly-(D,L-lactide-co-glycolide) has been used very successfully as a nanosystem for targeted delivery of drugs and other molecules. Up to now, poly -(D,L-lactide-co-glycolide)-based nanosystem have been reported with least toxicity, as it undergoes hydrolysis and produce biocompatible metabolites, lactic acid and glycolic acid. However, there has been recently published one report proposing that surface coating induces the toxicity of polymeric NPs towards human-like macrophages^[^^79^^]^. 

## DISCUSSION

Currently, the toxicity of engineered NPs is assessed with a number of approaches. Among them, the most beneficial one in term of cost and time saving are the *in vitro *studies. However so far, the *in vitro* studies in different laboratories have produced varying results. Cell viability is assessed most commonly by tetrazolium reduction assays, cell membrane integrity with LDH assay, immunohistochemistry biomarkers for apoptosis, and comet assay for genotoxicity. For intracellular localization of NPs, electron microscopy is employed^[^^46^^,^^80^^]^. To detect viable cells, compounds such as MTT, MTS, XTT, and WST-1 are used. MTT being a positive compound readily enter the viable eukaryotic cells while negative compounds such as MTS, XTT, and WST-1 do not permeate cells rapidly. Among all, the MTT tetrazolium assay has been widely adopted in laboratories for evaluation of cell toxicity (Table 1). MTT and other assay techniques require incubation of a reagent with cell culture. Viable cells convert the reagent into a color or a fluorescent product, which is then detected on a plate reader. In case of non-viable cells, the ability to convert reagent into a color or fluorescent product is lost^[^^81^^,^^82^^]^. Due to unique physicochemical properties, NPs interact with assay components or interfere with read out and may produce variable results, as noticed for carbon nanomaterials^[^^83^^]^. NPs induce the formation of ROS, which may affect the mitochondrial enzymes and subsequently the final read out^[^^46^^]^. 

Moreover, it has been reported in literature that the absorption spectrum of reduced MTT depends on pH^[^^84^^]^, and metal ions interfere with reduction reaction of MTT^[^^85^^]^. However more recently, a real-time cell-microelectronic sensing technique, with minimum interference, has been employed for evaluating NPs-induced cytotoxic effects^[^^86^^]^. Furthermore, due to inherent optical properties, NPs present in the reaction mixture or on cell surfaces may directly interfere in the read out by increasing the light absorption as evident for sodium titanate NPs^[^^87^^,^^88^^]^. Substantial quantity of LDH is released from the cytosol after cellular necrosis^[^^89^^]^. LDH assay has been used to determine the cytotoxicity of many NPs produced from silica, iron oxide, titanium oxide, and zinc oxide^[^^33^^,^^49^^,^^90^^-^^93^^]^. However, there is a concern in scientific community about the consistency of LDH assay. As reported by Nachlas *et al.*^[^^94^^]^, LDH activity is significantly decreased under low pH conditions while high pH destabilizes it.

ELISA is used to detect inflammatory biomarkers in cell culture. To estimate cell inflammation, chemokines IL-8, TNF-α, and IL-6 are used as biomarkers^[^^88^^,^^90^^]^. However, it has been noticed that cytokines may be adsorbed on NPs surfaces and interfere with the results of enzymatic immunoassays as observed in case of IL-8 for carbon nanomaterials and IL-6 for metals oxide NPs^[^^83^^,^^95^^]^. The sterility of NPs needs to be considered, as most of the NPs are manufactured in an unsterile environment having either bacteria or endotoxins, so the level of inflammatory markers may be altered by utilizing unsterilized NPs^[^^96^^,^^97^^]^. 

Due to unique physicochemical characteristics of NPs, inconsistent toxicological data have been generated even from well-established *in vitro* models. Physico-chemical properties of NPs, high adsorption capacity, alteration of pH, optical properties, surface charge, dissolution, magnetism, and catalytic behavior may either interfere with assay materials or detection system^[^^88^^]^. The risk of using cell lines for toxicity studies has been documented by Donaldson *et al.*^[^^98^^]^. These authors are of the opinion that in *in vitro* conditions, cell experiences a different toxic response as likely to be seen *in vivo*. Moreover, in laboratory, carcinoma cell lines having a different pathophysiology from normal cells are usually employed for *in vitro* toxicity testing of NPs, and the toxicological data derived from using such type of cell lines might be conflicting with that of normal cells^[^^99^^]^. 

In addition, NPs derived from certain bioceramic substances such as hydroxyapatite have been widely used in medicine since long ago, particulary in contact with bone. Also, hydroxyapatite has been reported to possess high biocompatibility towards bone cells. One research study has suggested that after intravenous injection, nanohydroxyapatite carrys no bioaccumul-ative toxicity in rabbit^[^^100^^,^^101^^]^. However, comprehensive toxicity data regarding nanohydroxyapatite is still lacking.

NPs are being used in a variety of sectors, and their use is increasing. Based on multiple uses in many areas, human exposure to NPs, both intentionally and unintentionally, is inevitable. Before being considered for human application, all nanoproducts are subjected to toxicological studies and for this purpose, several experimental studies are carried out. To meet this regulatory requirement, some toxic effects of nanomaterials have been evaluated, but according to reports, the toxicological data derived so far is conflicting and inconsistent. Toxicological studies provide a base for the protection of both human and environment. Therefore, on the basis of available experimental models, it may be difficult to list some of the more valuable NPs as more toxic to biological systems and vice versa. Considering the potential applications of NPs in many fields and to address the knowledge gap, the relevant toxic effects of NPs should be assessed by utilizing internationally agreed free of bias *in vivo* toxicological models, targeting the vital systems. However, in addition to all, we are of the opinion that designing, adapting, and validating such new models in future for toxicity testing, route of exposure, coating material and sterility of NPs, and type of cell cultures need to be carefully considered. 

**Table 1:** *I* *n vitro/in vivo* studies on toxicity of various types of NPs

**NPs and ** **size (nm)**	**Concentration and ** **exposure duration**	**Species/cell culture**	**Assay ** **technique**	**Result**	**Ref.**
Aluminum oxide (8-12)	1-10 µM 24 h	HBMVECs	MTT DHE	Cell viability ↓ Mitochondrial function ↓ Oxidative stress ↑ Alter proteins expression of the BBB	^[16]^
Aluminum oxide (50-80)	10, 50, 100, 200, 400 µg/mL 24 h	Mammalian cells	EZ4U	No significant toxic effect on cell viability	^[17]^
Aluminum oxide (160)	25-40 µg/mL 12 h	HMSC	MTT	Cell viability ↓	^[18]^
					
Aluminum oxide (30-40)	500-2000 mg/kg 72 h	Rat blood cells	Comet Micronucleus	Dose-dependent genotoxicity	^[19]^
Aluminum oxide (50)	0-5000 µg/mL 2 h	MLCL	Comet	DNA damage	^[20]^
					
Copper oxide (50)	10, 25, 50 µg/mL 24 h	Human lung epithelial cells	MTT LDH	Cell viability ↓ LDH ↑ Lipid peroxidation ↑	^[30]^
MWCNTs (20)	0.002-0.2 µg/mL 4 days	Lung cancer cells	MTT	Cell viability ↓	^[58]^
					
					
SWCNT (800)	0-400 µg/mL 10 days	HACECs NHBECs	Clonogenic	Cell death	^[59]^
SWCNTs (10-30)	40 and 200 µg/mouse, 1 mg/mouse, 90 days	*in vivo*	Commercial kits	LDH ↑ AST ↑ ALT ↑	^[61]^
Fullerenes (178)	1 ng/mL 80 days	CHO HELA HEK293	Micronucleus test	DNA strand breakage Chromosomal damage	^[67,68]^
Silica (15-46)	10-100 µg/mL 48 h	Human bronchoalveolar carcinoma cells	DCFH-DA Commercial kit	ROS ↑ LDH ↑ Malondialdehyde ↑	^[73]^
Silica (43)	25-200 µg/mL 3-24 h	Hepatocellular carcinoma cells (HepG2)	DCFH-DA 5,5,6,6-tetraethyl-benzimidazo-lylcarbo-cyanide iodine	ROS ↑ Mitochondrial damage Oxidative stress ↑	^[76]^
Silver (15-100)	10-50 µg/mL 24 h	BRL 3A	LDH MTT Glutathione DCFH-DA	Cell viability ↓ LDH ↑ ROS ↑	^[33]^
Silver (30-50)	0-20 µg/mL 24 h	Human alveolar cell line	MTT DCFH-DA	Cell viability ↓ ROS ↑	^[34]^
					
Silver (20-40)	---	Human leukemia cell line	WST-1 LDH	Cell viability ↓ LDH ↑	^[35]^
					
Zinc oxide (50-70)	11.5 µg/mL 24 h	Human colon carcinoma cells	ELISA Flow-cytometry	Oxidative stress↑ Cell viability↓Inflammatory biomarkers	^[1]^
Zinc oxide (307-419)	10-100 µg/mL 24-48 h	Human cervix carcinoma cell line (HEp-2)	Comet micronucleus test MTT	DNA damage Cell viability ↓	^[42]^
Zinc oxide (30-70)	14-20 µg/mL 12 h	*in vivo*	MTT Comet DCFH-DA	Cell viability ↓ DNA damage ROS Apoptosis	^[43]^
Zinc oxide (50)	0-100 µg/mL 24 h	Human hepatocytes HEK 293 cell line	MTT Comet	DNA damage Cell viability ↓ Oxidative stress Mitochondrial damage	^[40]^
Zinc oxide (<20)	100 µg/mL	Human bronchial epithelial cells	-	Cell viability ↓ Oxidative stress ↑ LDH release	^[37]^
Iron oxide (30)	25-200 µg/mL 2 h	Murine macrophage cells	MTT	Cell viability ↓	^[44]^
					
Iron oxide (100-150)	0.1 mg/ mL 7 days	Human macrophages	MTS	Cell viability ↓	^[49]^
Iron oxide (13.8)	123.52 µg/mL 12 h	Human hepatocellular carcinoma cells	MTT	Cell viability ↓	^[50]^
Iron oxide (20)	0.1 mg/mL 2 days	Rat mesenchymal stem cells	MTS	Cell viability ↓	^[51]^
Titanium oxide (160)	1800 µg/mouse 10 days	*in vivo*	Comet micronucleus test	DNA damage Genotoxicity	^[53]^
Titanium oxide (<100)	10-50 µg/mL 6-24 h	Human lung cells	ELISA Trypan blue DCFH-DA	Oxidative stress ↑ DNA adduct -formation Cytotoxicity ↑	^[54]^

HBMVECs, Human brain micro vascular endothelial cells; DHE, Dihydroethidium; BBB, blood- brain- barrier; HMSC, Human mesenchymal stem cells; MLCL, Mouse lymphoma cells line; LDH, Lactate dehydrogenase; MWCNTs, Multi- walled carbon nano tubes; SWCNTs: Single walled carbon nano tubes; HACECs, Human alveolar carcinoma epithelial cell line; NHBECs, Normal human bronchial epithelial cell line; AST: Aspartae transaminase; ALT, Alanine transaminase; CHO, Chinese Hamster ovary cells; HELA, Human epidermoid-like-carcinoma cells; HEK: Human embryonic kidney cells; DCFH-DA, Dichlorodihydrofluorescein diacetate; ROS, Reactive oxygen species; BRL 3A, Buffalo rat liver cells. ↑ = increase and ↓= decrease.

Moreover, the US FDA as a public health agency has also recently taken into account this important issue of toxic effects associated with products containing NPs and do not consider them either totally safe or harmful for human use, and each product will be subjected to regulation.
